# Indigenous language and inequitable maternal health care, Guatemala, Mexico, Peru and the Plurinational State of Bolivia

**DOI:** 10.2471/BLT.18.216184

**Published:** 2018-10-31

**Authors:** Nancy Armenta Paulino, María Sandín Vázquez, Francisco Bolúmar

**Affiliations:** a Public Health Unit, Faculty of Medicine, University of Alcalá, Crtra Madrid-Barcelona Km 33.6, Alcalá de Henares, 28871, Spain.

## Abstract

Latin America and the Caribbean still have high maternal mortality rates and access to health care is very uneven in some countries. Indigenous women, in particular, have poorer maternal health outcomes than the majority of the population and are less likely to benefit from health-care services. Therefore, inequities in maternal health between different ethnic groups should be monitored to identify critical factors that could limit health-care coverage. In adopting the United Nations’ sustainable development goals, governments have committed to providing equitable and universal health coverage. It is, therefore, the right time to assess ethnic disparities in maternal health care. However, finding a standard method of identifying ethnicity has been difficult, because ethnicity involves several features, such as language, religion, tribe, territory and race. In this study, spoken indigenous language was used successfully as a proxy for ethnicity to detect inequities in maternal health-care coverage between indigenous and non-indigenous populations in four Latin American countries: Guatemala, Mexico, Peru and the Plurinational State of Bolivia. Although, quantifying ethnic inequities in health care is just a starting point, this quantification can help policy-makers and other stakeholders justify the need for monitoring these inequities. This monitoring is essential for designing more culturally appropriate programmes and policies that will reduce the risks associated with maternity among indigenous woman. As long as inequities persist, identifying them is an important step towards their elimination.

## Résumé

L'Amérique latine et les Caraïbes continuent d'afficher des taux de mortalité maternelle élevés et dans certains pays, l'accès aux soins de santé est très inégal. Les femmes autochtones, en particulier, sont dans un plus mauvais état de santé maternelle que la majorité de la population et sont moins susceptibles de bénéficier des services de santé. Il convient donc de suivre les inégalités relatives à la santé maternelle entre les différents groupes ethniques pour identifier les facteurs déterminants qui peuvent limiter la couverture sanitaire. En adoptant les objectifs de développement durable des Nations Unies, les gouvernements se sont engagés à fournir une couverture sanitaire équitable et universelle. Il est donc temps d'évaluer les disparités ethniques en matière de soins de santé maternelle. Il s'est néanmoins avéré difficile de trouver une méthode standard permettant de définir l'appartenance ethnique, car cette dernière implique plusieurs caractéristiques, telles que la langue, la religion, la tribu, le territoire et la race. Dans cette étude, la langue autochtone parlée a été utilisée avec succès en tant qu'indicateur d'appartenance ethnique pour détecter les inégalités concernant la couverture des soins de santé maternelle entre les populations autochtones et non autochtones de quatre pays latino-américains: l'État plurinational de Bolivie, le Guatemala, le Mexique et le Pérou. Bien que la quantification des inégalités ethniques en matière de soins de santé ne soit qu'un point de départ, elle peut aider les responsables politiques et d'autres parties prenantes à justifier la nécessité d'un suivi de ces inégalités. Ce suivi est essentiel pour concevoir des programmes et des politiques mieux adaptés à la culture des populations et réduire ainsi les risques associés à la maternité chez les femmes autochtones. Tant que des inégalités persistent, les identifier est une étape importante vers leur élimination.

## Resumen

América Latina y el Caribe siguen teniendo altas tasas de mortalidad materna y el acceso a la atención sanitaria es muy desigual en algunos países. Las mujeres indígenas, en particular, tienen peores resultados en salud materna que la mayoría de la población y menos probabilidades de beneficiarse de los servicios de atención sanitaria. Por tanto, deben vigilarse las desigualdades en temas de salud materna entre los diferentes grupos étnicos para determinar los factores críticos que podrían limitar la cobertura de la atención sanitaria. Al adoptar los objetivos de desarrollo sostenible de las Naciones Unidas, los gobiernos se han comprometido a proporcionar una cobertura sanitaria equitativa y universal. Por tanto, es el momento adecuado para evaluar las disparidades étnicas en la atención sanitaria materna. Sin embargo, ha sido difícil encontrar un método estándar para identificar la etnia, pues esta tiene varias características, como el idioma, la religión, la tribu, el territorio y la raza. En este estudio, el idioma indígena hablado se utilizó con éxito como indicador de la etnicidad para detectar las desigualdades en la cobertura de la atención sanitaria materna entre las poblaciones indígenas y no indígenas en cuatro países de América Latina: el Estado Plurinacional de Bolivia, Guatemala, México y Perú. Aunque la cuantificación de las inequidades étnicas en la atención sanitaria es solo un punto de partida, esta cuantificación puede ayudar a los responsables de la formulación de políticas y a otros interesados a justificar la necesidad de monitorizar estas inequidades. Esta monitorización es esencial para diseñar programas y políticas culturalmente más adecuadas que reduzcan los riesgos asociados con la maternidad entre las mujeres indígenas. Mientras persistan las desigualdades, identificarlas es un paso importante hacia su eliminación.

## ملخص

لا تزال أمريكا اللاتينية ومنطقة البحر الكاريبي تعاني من ارتفاع معدلات وفيات الأمهات، كما أن الحصول على الرعاية الصحية متفاوت للغاية في بعض الدول. تعاني السيدات من أهل البلاد الأصليين، على وجه الخصوص، من مستويات صحية للأمومة أكثر فقراً من غالبية السكان، كما تقل لديهن احتمالية الاستفادة من خدمات الرعاية الصحية. لذلك، فإن عدم المساواة في صحة الأم بين المجموعات العرقية المختلفة يجب أن تخضع للمراقبة بهدف تعريف العوامل الأساسية التي يمكن أن تحد من تغطية الرعاية الصحية. وفي سبيل اعتماد أهداف التنمية المستدامة في الأمم المتحدة، التزمت الحكومات بتوفير العدالة والتغطية الصحية الشاملة. ولذلك، فإن هذا هو الوقت المناسب لتقييم التفاوتات العرقية في الرعاية الصحية للأمهات. ومع ذلك، فإن العثور على طريقة قياسية تحديد العرق كانت، ولا تزال صعبة، لأن العرق ينطوي على العديد من الخصائص، مثل اللغة والدين والقبيلة والمنطقة والأصل. تم في هذه الدراسة تم استخدام لغة السكان الأصليين المنطوقة بنجاح كمؤشر للعرق للكشف عن حالات عدم المساواة في تغطية الرعاية الصحية للأمهات، بين السكان الأصليين وغير الأصليين في أربعة بلدان في أمريكا اللاتينية: المكسيك، وبوليفيا (دولة متعددة القوميات)، وبيرو، وغواتيمالا. على الرغم من أن القياس الكمي لحالات عدم المساواة العرقية في الرعاية الصحية هو مجرد نقطة بداية، فإن هذا القياس الكمي يمكن أن يساعد صانعي السياسات وأصحاب المصلحة الآخرين في تبرر الحاجة إلى مراقبة هذه الحالات. هذا الرصد ضروري لوضع برامج أكثر ملاءمة ثقافياً، وسياسات تحد من المخاطر المرتبطة بالأمومة بين النساء من أهل البلاد الأصليين. طالما استمرت حالات عدم المساواة، فإن تحديدها يعتبر خطوة هامة نحو القضاء عليها.

## 摘要

拉丁美洲和加勒比地区的孕产妇死亡率仍然很高，一些国家的医疗保健服务水平显著参差不齐。特别是土著妇女中孕产妇的健康结果比孕产妇中大多数人群的健康结果差，并且土著妇女从医疗保健服务中受益的可能性更小。因此，不同民族群体之间孕产妇健康的不平等现象应得到监测，以确定可能限制医疗保健覆盖的关键因素。在采用联合国可持续发展目标时，各国政府致力于提供平等、全面的健康覆盖。因此，现在是评估孕产妇医疗保健中民族差异的适当时机。然而，找到一种确定民族划分的标准方法始终很难，因为民族涉及一些诸如语言、宗教、部落、领土和种族的特征。本研究成功将使用土著语口语作为民族的代表，发现土著和非土著人口之间孕产妇医疗保健覆盖的不平等现象存在于以下四个拉丁美洲国家：危地马拉、墨西哥、秘鲁和玻利维亚（多民族国）。虽然量化医疗保健中的种族不平等只是一个起点，但此类量化可以帮助政策制定者和其他利益相关者证明监测这些不公平现象的必要性。这种监测对于设计出文化上更为合适的方案和政策至关重要，它们将减少土著妇女与孕产妇相关的风险。只要不公平现象持续存在，识别其存在是消除不公平现象的重要一步。

## Резюме

Уровень материнской смертности в странах Латинской Америки и Карибского бассейна сохраняется высоким, а доступ к медико-санитарным услугам в некоторых странах распределен очень неравномерно. В частности, женщины-представители коренных народов имеют худшие показатели материнского здоровья и исходы материнства, чем большинство населения, и для них вероятность воспользоваться услугами здравоохранения оказывается ниже. Следовательно, неравенство в сфере охраны материнского здоровья в различных этнических группах необходимо отслеживать для выявления критических факторов, которые могут ограничивать охват населения услугами здравоохранения. Внедряя предложенные ООН цели устойчивого развития, правительства берут на себя обязательства обеспечивать всеобщий и равный доступ к услугам здравоохранения. Следовательно, пришло время оценить этническое неравенство в предоставлении услуг по охране материнского здоровья. Однако найти стандартный метод определения этнической принадлежности сложно, так как это понятие включает несколько факторов, таких как язык, религия, племенная принадлежность, проживание на определенной территории и расовая принадлежность. В данном исследовании язык коренных народов в повседневном общении успешно использовался в качестве замены фактора этнической принадлежности при выявлении неравенства в предоставлении услуг охраны материнского здоровья между коренной и некоренной популяциями стран Латинской Америки: Боливии (многонациональное государство), Гватемалы, Мексики и Перу. Несмотря на то что количественная оценка этнического неравенства в здравоохранении — это всего лишь начальная точка, она может оказаться полезной для лиц, принимающих стратегические решения, и других участников процесса при обосновании необходимости мониторинга такого неравенства. Такой мониторинг крайне важен для разработки программ и стратегий, учитывающих культурные особенности, которые бы снизили связанные с материнством риски для женщин-представителей коренных национальностей. Пока неравенство существует, выявление конкретных случаев — это первый шаг к его устранению.

## Introduction

Countries in Latin America and the Caribbean have some of the highest adolescent pregnancy rates in the world and adolescent pregnancies are more common among uneducated, poor and indigenous women. In these countries, women with socioeconomic disadvantages are more likely to postpone seeking care and experience delays in accessing services and receiving adequate health care.^1^^,^^2^ These difficulties highlight the challenges still faced in these countries in improving maternal health.^1^^,^^3^

Indigenous women form one of the most vulnerable groups in these countries: they experience substantially worse maternal health outcomes than the majority of the population and are less likely to benefit from services.^1^^,^^4^ In addition, they are more likely than other women to experience social and economic exclusion and to die during pregnancy or childbirth.^5^^,^^6^ Indigenous populations are adversely affected by a combination of different social determinants of health, such as poverty, limited education, disadvantageous gender roles and cultural factors. Table 1 lists differences in some of these social determinants between indigenous and non-indigenous people in four Latin American countries.^7^^,^^8^ In addition, health disparities between different ethnic groups may also reflect the effect of discrimination on access to health services, or on the quality of the care provided.^1^^,^^9^^,^^10^

**Table 1:** Characteristics of indigenous and non-indigenous people and maternal health care, Guatemala, Mexico, Peru and the Plurinational State of Bolivia, 2010 and 2015

Characteristic	Country			
	Guatemala	Mexico	Peru	Plurinational State of Bolivia
**Maternal mortality ratio in 2015, per 100 000 live births**	88	38	68	206
**Maternal health issues in 2015**	(i) indigenous women had a maternal mortality ratio three times that in non-indigenous women; (ii) only 30% of indigenous women had a skilled birth attendant; and (iii) the proportion of women with an unmet need for contraception was four times higher in the poorest quintile than the richest	(i) pregnant women with private insurance had more antenatal consultations and received higher-quality services than women with public or no insurance; (ii) a low educational level increased a woman’s risk of dying from eclampsia or haemorrhage; and (iii) women with pregnancy complications experienced delays because of ineffective triage	(i) the maternal mortality ratio in some mainly indigenous regions was more than six times higher than in the national capital; (ii) the difference between the poorest and richest quintiles in the proportion of women who had a skilled birth attendant was 32 percentage points; and (iii) in some areas, the advanced equipment needed for emergency obstetric care was available only in provincial capitals	(i) the maternal mortality ratio was one of the highest in the world; (ii) the difference between the poorest and richest quintiles in the proportion of women who had at least four antenatal visits was greater than 20 percentage points; and (iii) the difference between rural and urban women in the proportion who had a skilled birth attendant was 26 percentage points
**Indigenous population in 2010**	5 881 009	16 933 283	7 021 271	6 216 026
**Indigenous people as a proportion of the population in 2010, %**	41.0	15.1	24.0	62.2
**Ethnic inequities in 2010**				
Proportion living on less than US$ 4 per day, %				
Indigenous people	77	40	32	44
Non-indigenous people	49	23	16	20
Proportion educated to lower than primary level, %				
Indigenous people	43	48	52	41
Non-indigenous people	20	33	35	22
Proportion living in rural areas, %				
Indigenous people	ND	46	47	52
Non-indigenous people	ND	19	18	13

ND: not determined.

Data sources: The World Bank.^7^^,^^8^

Given these disparities, it is both useful and necessary to monitor inequities in maternal health between different ethnic groups. Monitoring would help quantify differences between groups and identify critical factors that limit the coverage of care. Governments, health-care organizations and other key actors could then focus research on problematic areas to determine their cause. Subsequently, policies, programmes and practices could be changed to benefit the health of indigenous women and to ensure that resources are allocated efficiently.^11^

By accepting the United Nations’ sustainable development goals (SDGs), governments have committed themselves to continuing efforts to reduce maternal mortality and inequities in maternal health, both within and between countries. The agenda of the SDGs provides a major impetus for establishing or strengthening systems for monitoring health inequalities and calls for the production of “data disaggregated by income, gender, age, ethnicity, disability and other relevant characteristics.”^12^ This is, therefore, the right time to assess disparities in maternal health care between different ethnic groups. However, quantifying the influence of ethnicity on health inequities is not an easy task. Ethnicity is not defined by fixed or easily measurable characteristics; it is instead considered a subjective and contextual concept that involves several dimensions, such as language, religion, tribe, territory and race.^13^^,^^14^ The main obstacles are a lack of disaggregated data and the difficulty of identifying ethnicity in a consistent or standardized way across countries. Previous surveys carried out in several countries have used a heterogeneous set of questions to capture ethnicity and there have even been differences between surveys repeated in the same context in different years.^15^^–^^18^

In previous studies of the size of the indigenous population in Latin America and the Caribbean, the most common criterion used for identifying ethnicity is spoken indigenous language. Questions about language have been included in censuses and national surveys for many years.^17^^,^^19^^–^^22^ From a social perspective too, spoken indigenous language has been considered a marker of ethnicity because it is a manifestation of people’s attachment to their culture.^17^^,^^19^^,^^23^ Consequently, this criterion may be useful for studying variations in health inequities between different ethnic groups. Here we report on how the criterion of spoken indigenous language can be used as a proxy for ethnicity in investigations of inequities in maternal health care coverage between indigenous and non-indigenous populations.

## Key concepts

### Ethnic and indigenous groups

An ethnic group is defined as a collectively that identifies itself, and it is identified by others, with regard to certain common elements, such as language, religion, tribe, nationality, race or a combination thereof, and whose members share a common feeling of identity.^16^^,^^24^^,^^25^ An indigenous group is a particular form of ethnic group: its members have an established history in a particular territory and have a common language and culture.^17^^,^^18^^,^^24^ At least four elements should be taken into account in defining indigenous peoples: (i) recognition of identity; (ii) common origin (iii) territoriality; and (iv) the linguistic-cultural dimension. The first element refers to the sense of belonging to a group, the second refers to the idea of coming from common ancestors, the third recognizes traditional occupation of a specific territory, and the fourth is linked to an attachment to a culture, language, worldview and way of life.^17^^,^^21^

### Ethnicity and language

Ethnic group expresses its culture and social identity through language, because language is intimately linked to mental and ideological processes and to the perception of internal and external worlds. Language is a fundamental point of reference by which an ethnic group finds its own identity. Many indigenous cultures have traditional knowledge that is transmitted only orally.^4^^,^^22^^,^^23^^,^^26^

Despite the problem of how to deal with data on multilingual individuals, people who report speaking an indigenous language are highly likely to be members of the indigenous group that speaks that language, because language is more than simply a means of communication. Language is also a central element of culture and of the process of socialization.^4^^,^^19^^,^^23^^,^^26^ Language is therefore important for studying health care in indigenous groups. Language can be used as a proxy for membership of an indigenous group and is a strong determinant of access to health care.^20^^,^^27^ The presence of a language barrier has been closely linked to the limited access to health care that results from being unable to communicate with health-care personnel. Several studies have documented that poor health outcomes are more likely when there are language and cultural barriers between patients and health-care providers.^28^^,^^29^ Language barriers may also influence patients’ perceptions of the quality of care. Conversely, it is also possible to use the criterion of language to indirectly investigate differences in health care associated with these barriers.

## Measuring inequities by ethnicity

**Fig. 1:**
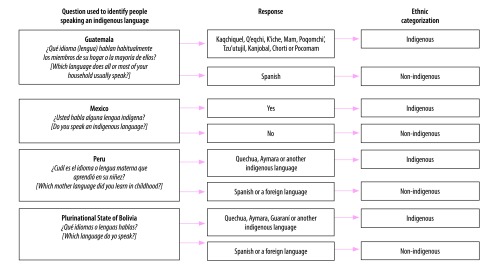
Survey questions used to identify people speaking an indigenous language, Guatemala, Mexico, Peru and the Plurinational State of Bolivia, 2008, 2009 and 2015

Note: The questions listed were used in the 2008 Demographic and Health Survey in Bolivia, the 2008–2009 National Survey on Maternal and Child Health in Guatemala, the 2015 National Survey of Children and Women in Mexico and the 2015 Demographic and Health Survey in Peru.^30^^–^^32^

Studying how inequities in health vary according to ethnicity involves dividing a study population into appropriate groups. However, how subgroups within a population are defined may depend on the method of data collection, the data available and the population’s characteristics. In the past, most surveys performed in Latin America and the Caribbean asked whether people spoke an indigenous language, what language they spoke or which language was spoken most often in their homes. Examples of the questions asked in four Latin American countries are shown in Fig. 1. We believe that using the criterion of primarily speaking an indigenous language enables us to identify a group of women who share a culture related to maternity, who could experience a language barrier and who could suffer discrimination, all of which may affect maternal health care.

### Selecting indicators

**Fig. 2:**
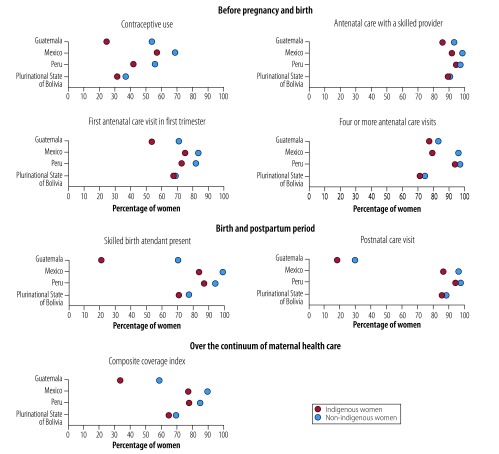
Maternal health care coverage, by ethnicity, Guatemala, Mexico, Peru and the Plurinational State of Bolivia, 2008, 2009 and 2015

Notes: For each indicator, coverage was defined as the percentage of women who received the intervention among those who needed it. The composite coverage index is the weighted average of coverage of the six other indicators illustrated. Data for this figure were derived from the 2008 Demographic and Health Survey in Bolivia, the 2008–2009 National Survey on Maternal and Child Health in Guatemala, the 2015 National Survey of Children and Women in Mexico and the 2015 Demographic and Health Survey in Peru.^30^^–^^32^

Another essential consideration in evaluating health inequities is selecting the most appropriate indicators. For maternal health care, we believe that any analysis should consider women’s health-care coverage across the continuum of care from before pregnancy through to pregnancy, childbirth and the postpartum period. However, when viewed from the perspective of the continuum of care, every phase is important and, if possible, a composite index should be formed from all indicators monitored. Fig. 2 gives examples of the indicators used in previous studies. In addition, reducing mortality also depends on high care coverage and on the quality of the services provided.^33^ Nevertheless, the final decision on which indicators to monitor depends on the context and national and local needs.

### Case studies

We looked at ethnic inequities in maternal health care in four Latin American countries where a substantial proportion of the population is indigenous and where the maternal mortality ratio is high: Guatemala, Mexico, Peru and the Plurinational State of Bolivia. We obtained data from Demographic and Health Surveys and Multiple Indicator Cluster Surveys.^30^^–^^32^ When we used spoken indigenous language as a proxy for ethnicity, we found differences in maternal health care between different ethnic groups in all four countries despite efforts made over the last two decades to reduce maternal mortality in response to the millennium development goals (Fig. 2).^3^ In most cases, differences between ethnic groups were significant. However, ethnic differences in maternal health-care coverage varied substantially between countries and indicators. Differences were most apparent in the first and last stages of the continuum of care. The indicators that demonstrated the most substantial ethnic differences across all four countries were contraceptive use and the presence of a skilled birth attendant. These are the indicators that interventions should be focused on. The largest gaps in care for all indicators were in Guatemala and the smallest were in the Plurinational State of Bolivia (Fig. 2).

Although differences in the level of maternal health care coverage between ethnic groups may be due to differences in sociodemographic characteristics,^34^ reducing inequities between ethnic groups is more complex than simply modifying these characteristics. Only improving living conditions is not enough, because other social and cultural factors also have an influence in indigenous populations.^35^^–^^38^ In 2018, a study of inequities in maternal and child health interventions between ethnic groups found that, although they had decreased recently in countries such as Guatemala, Mexico and the Plurinational State of Bolivia, differences were still evident after adjustment for wealth, educational level and place of residence.^15^

## Discussion

The main advantages of using spoken language as a demographic characteristic are that it is an objective variable and that it is fixed. Moreover, to a certain extent spoken language is independent of the person's view of her- or himself and will, therefore, not change over time. In contrast, other characteristics, such as a person’s self-identification, depend on the person being recognized as indigenous and may be influenced by negative prejudices or cultural empathy.^19^^,^^39^ However, the main disadvantage of using language as a proxy for ethnicity is that the use of indigenous languages is gradually decreasing, particularly among the younger generation and urban populations. Therefore, such use will become increasingly difficult to base ethnic identity on spoken language, although such a proxy will still be useful in areas where groups are mainly monolingual.^17^^,^^19^^,^^21^^,^^39^ The gold standard would be to combine several attributes, such as language, self-identification and geographical location, as this would improve acuracy.^17^^,^^21^^,^^22^ However, this information is not always available.

In both quantitative and qualitative studies, speaking an indigenous language has been identified as one factor that influences coverage of maternal care services. Women who speak an indigenous language are less likely to have an institutional delivery and are more likely to attend fewer than four prenatal visits. Moreover, a smaller proportion of these women use modern contraceptives. In addition, the maternal mortality rate is higher in some areas where a large proportion of the population speaks an indigenous language.^40^^–^^45^ These findings are consistent with the low level of coverage of maternal care services observed among indigenous women in the countries we studied.

Using spoken indigenous language as a proxy for ethnicity enabled us to identify ethnic inequities in all countries analysed. Our findings are in line with those recently published, except in the Plurinational State of Bolivia, where those researchers observed greater inequities in maternal health care coverage when the criterion of self-identification was used as a proxy for ethnicity.^15^ The contrast between our findings and this previous study highlight two critical factors that should be considered when evaluating ethnic inequities. First, the method used to determine ethnicity can affect the magnitude of the inequity observed. Second, good understanding of the social context in a country is essential for accurately interpreting findings and for selecting the most appropriate proxy for ethnicity in that context. For example, in the Plurinational State of Bolivia, current social attitudes towards the indigenous population may increase people’s willingness to identify themselves as indigenous. In contrast, in some situations where discrimination and exclusion are common, people may not want to recognize themselves as indigenous.^17^^,^^19^^,^^46^^,^^47^

Our findings confirm that indigenous people are vulnerable to inequities in health care. Therefore, efforts should be made both locally and nationally to provide data disaggregated by ethnicity, because the lack of such data could obscure inequities that may lie behind the averages. Historically, the indigenous population in Latin America and the Caribbean has been invisible statistically, because few data from the region have been disaggregated by ethnicity.^4^^,^^17^

Future studies of ethnic inequities in indigenous populations should: (i) investigate the heterogeneity of the indigenous population; (ii) verify study findings using another criterion for identifying ethnicity; (iii) analyse trends in inequities over time; and (iv) evaluate other indicators of coverage across the continuum of maternal health care. Regardless of the criteria used to monitor ethnic inequities, transparency is needed about why the criteria have been used and about how ethnicity has been categorized if we are to understand the context and scope of a study’s findings. Moreover, we should be cautious about comparisons with other studies and about generalizing a study’s findings, because the observed magnitude of any inequity could be altered using a different criterion to identify ethnicity.

In conclusion, quantifying ethnic inequities in health care is just a starting point. Awareness of these inequities can help policy-makers and other stakeholders justify the need for monitoring and the use of spoken indigenous language as a criterion can be useful. Moreover, monitoring inequities is essential for designing more culturally appropriate programmes and policies that will reduce the risks associated with maternity among indigenous woman. As long as inequities persist, identifying them is an important step towards their elimination.
